# Limitation of Grassland Productivity by Low Temperature and Seasonality of Growth

**DOI:** 10.3389/fpls.2016.01130

**Published:** 2016-07-27

**Authors:** Astrid Wingler, Deirdre Hennessy

**Affiliations:** ^1^School of Biological, Earth and Environmental Sciences, University College Cork, CorkIreland; ^2^Teagasc-The Agriculture and Food Development Authority, Moorepark Animal & Grassland Research and Innovation CentreFermoy, Ireland

**Keywords:** brassinosteroids, gibberellins, grass breeding, growth modeling, perennial ryegrass (*Lolium perenne* L.), phytohormones, seasonality, winter persistence

## Abstract

The productivity of temperate grassland is limited by the response of plants to low temperature, affecting winter persistence and seasonal growth rates. During the winter, the growth of perennial grasses is restricted by a combination of low temperature and the lack of available light, but during early spring low ground temperature is the main limiting factor. Once temperature increases, growth is stimulated, resulting in a peak in growth in spring before growth rates decline later in the season. Growth is not primarily limited by the ability to photosynthesize, but controlled by active regulatory processes that, e.g., enable plants to restrict growth and conserve resources for cold acclimation and winter survival. An insufficient ability to cold acclimate can affect winter persistence, thereby also reducing grassland productivity. While some mechanistic knowledge is available that explains how low temperature limits plant growth, the seasonal mechanisms that promote growth in response to increasing spring temperatures but restrict growth later in the season are only partially understood. Here, we assess the available knowledge of the physiological and signaling processes that determine growth, including hormonal effects, on cellular growth and on carbohydrate metabolism. Using data for grass growth in Ireland, we identify environmental factors that limit growth at different times of the year. Ideas are proposed how developmental factors, e.g., epigenetic changes, can lead to seasonality of the growth response to temperature. We also discuss perspectives for modeling grass growth and breeding to improve grassland productivity in a changing climate.

## Introduction

The growth response of forage grass species to temperature has been studied extensively since the 1970s. More recently, improved understanding of the growth physiology of grass has been gained, but knowledge is still lacking how the temperature response of grassland species can be improved to allow sustained growth throughout the year. Work with model species and recent progress in grass genetics can improve our understanding of the processes that determine growth and identify targets for breeding. Knowledge gained will also be important for grassland management, e.g., for deciding how long and extensively pastures should be grazed for maximum productivity. Developing models for grass growth in response to temperature may enable forecasting of growth dependent on season and temperature, and also how grassland productivity may be affected by climate change.

## Seasonal Patterns of Grass Growth

Temperature has a major impact on the growth of temperate forage grasses, such as perennial ryegrass (*Lolium perenne*). For example, soil temperature was identified as the main determinant of growth in the South of Ireland (Hurtado-Uria et al., 2013a; see Monitoring Grassland Productivity). While low rates of growth can occur at temperatures down to 0°C, leaf elongation of perennial ryegrass was shown to increase strongly at temperatures above 5°C (Peacock, 1975). More recent work (Nagelmüller et al., 2016) also demonstrates low rates of leaf elongation in winter wheat, summer barley and perennial ryegrass at temperatures down to 0°C, with an abrupt increase above 5°C. Minor, but significant genotype-specific differences were found for growth at low temperatures, which could be exploited in breeding.

In addition to environmental conditions, developmental factors determine the growth of grass: productivity is highest in late spring and early summer and declines later in the summer (Hurtado-Uria et al., 2013a). Although, productivity is determined not only by the growth of individual leaves, but also by leaf production/tillering, measurement of leaf elongation can provide useful physiological information about the processes that determine biomass production. Often, there is a distinct peak of leaf extension in spring (Peacock, 1975; Parsons and Robson, 1980; Davies et al., 1989). These seasonal effects are demonstrated by transfer from cold into warm conditions. For example, a growth spurt was found after transfer of Italian and perennial ryegrass to warm conditions in February, but growth was lower after transfer in mid-April (Davies et al., 1989). This shows that ryegrass has the highest potential for growth early in the year, whereas developmental factors limit the temperature response later in the season. Furthermore, mild frost in winter and early spring can stimulate compensatory dry matter (DM) production in perennial grasses (Østrem et al., 2010).

## Cold Acclimation and Winter Persistence

An important factor for grassland productivity is winter persistence. Even in mild climates loss of biomass can occur during the winter (e.g., Hennessy et al., 2006), and lack of persistence can also affect the relative abundance of species and cultivars after the winter, e.g., resulting in a loss of the biomass of clover, which is often grown with grass in mixed swards (Wachendorf et al., 2001) and loss of perennial ryegrass tillers (Hennessy et al., 2008).

The process of cold acclimation that results in winter hardiness has been well characterized in temperate forage grasses. Fructans, which accumulate during summer and autumn and peak in December (Pollock and Jones, 1979), can protect the plants against stress (Sandve et al., 2011) and serve as a carbon source for regrowth in spring (Pollock and Jones, 1979; Tamura et al., 2014). The C-repeat binding factor (CBF) dependent cold acclimation pathway that was originally identified in *Arabidopsis thaliana* is also active during cold acclimation in perennial ryegrass (Xiong and Fei, 2006). More recently, analysis of changes in the transcriptome of perennial ryegrass showed disruption of the circadian rhythm network during cold acclimation in a variety adapted to warmer climates (Abeynayake et al., 2015). Candidate gene association mapping identified alleles of genes associated with winter survival and spring regrowth, including a *CBF* gene (Yu et al., 2015).

Compared to forage grasses, the information about processes underlying cold acclimation is limited for white clover (*Trifolium repens*), which can show lack of persistence during the winter. White clover requires slightly higher temperatures for growth than perennial ryegrass, thus limiting its persistence in mixed swards at temperatures below 10°C (Collins and Rhodes, 1995). In addition to low temperature alone, shading by grass tillers has a negative impact on the clover content of mixed swards, but only in combination with low spring and winter temperatures (Wachendorf et al., 2001). In white clover carbon accumulates in the form of pinitol and sucrose in the stolons during winter (Turner and Pollock, 1998), and cold exposure results in the accumulation of vegetative storage proteins in the roots and stolons. These vegetative storage proteins have a role as nitrogen store, but they may also be involved in the cold acclimation process (Goulas et al., 2003).

There are trade-offs between cold acclimation and growth during the winter – the synthesis of compounds required for cold hardiness limits carbon availability for growth. As discussed by Parsons et al. (2013) growth of perennial grasses may be limited not by resource availability but by maximizing long-term fitness. Breeding for increased frost hardiness can therefore result in low growth rates, whereas cultivars with increased growth at low temperature may be more susceptible to damage by frost. Growth and frost hardiness therefore both need to be optimized together for future climatic conditions.

## Physiological Mechanisms Underlying Grassland Productivity During the Growing Season

In humid temperate climates, uptake of CO_2_ by grassland can continue throughout the year (Peichl et al., 2011), even at below-zero temperatures (Skinner, 2007), although solar radiation and daylength may be limiting photosynthesis during the winter months. Once light conditions become more favorable, growth can be actively restricted by processes that inhibit cell division and expansion at low temperature. Thus, instead of being source-limited, growth becomes sink-limited (Wingler, 2015). Hormone signaling pathways play an important role in this regulation, in particular gibberellic acid (GA) signaling. For *Arabidopsis* it has been shown that GA stimulates growth by targeting growth-inhibiting DELLA proteins for degradation. At low temperature, CBF-dependent cold acclimation reduces GA content, resulting in DELLA accumulation and growth inhibition (Achard et al., 2008).

Gibberellic acid also determines the growth of grass species; for example, it can promote leaf extension of perennial ryegrass (Stapleton and Jones, 1987). It was proposed that GA plays a role in the induction of the fructan-degrading enzyme fructan exohydrolase to promote growth after defoliation (Morvan et al., 1997). As carbohydrate content declines, expression of the gene for the GA activating GA3 oxidase increases and that of the gene for the inactivating GA2 oxidase decreases (Liu et al., 2015). It was therefore proposed that an interaction between sugar and GA metabolism is responsible for the growth response to defoliation. In an association mapping study it was demonstrated that polymorphism of a gene for the DELLA protein *GAI* (GA insensitive) can explain differences in leaf elongation in perennial ryegrass (Auzanneau et al., 2011), demonstrating that not just the synthesis of active GA, but also GA signaling is involved in the growth response.

Similar to GA, brassinosteroids stimulate cell division and expansion (Fridman and Savaldi-Goldstein, 2013), and brassinosteroid insensitivity results in increased cold tolerance in *Arabidopsis* (Kim et al., 2010). Recently, brassinosteroids have been shown to regulate GA production, thus integrating both hormone signaling pathways in *Arabidopsis* (Unterholzner et al., 2015). Work with the model *Brachypodium distachyon* demonstrated that brassinosteroid signaling also determines growth in a grass species (Thole et al., 2012) and that down-regulation of brassinosteroid signaling results in increased drought tolerance (Feng et al., 2015). However, the importance of brassinosteroids in temperature-dependent growth and possible interactions with GA have not been reported in grass species. Jasmonic acid inhibits growth in response to low temperature in dicot species, such as *Arabidopsis* (Wingler, 2015), and also seems to have a growth inhibitory effect in grasses, although its role is less well-explored than in dicots (Shyu and Brutnell, 2015).

## What Determines the Seasonality of Grass Growth?

In spring, grass has a higher capacity to respond to warm temperature than later in the season. Peacock (1975) proposed that developmental instead of environmental factors are responsible for these seasonal differences in grass growth. Later work provided some insight into the nature of these factors. Reproductive tillers were found to have higher rates of leaf extension than those that remain vegetative (Davies et al., 1989). This is in agreement with the finding that vernalization stimulates leaf extension (Stapleton and Jones, 1987). Seasonal differences in the growth response to GA that may explain the seasonality of growth were identified (Ball et al., 2012; Parsons et al., 2013): perennial ryegrass plants taken from the field in winter showed a stronger increase in DM production in response to treatment with GA than plants taken from the field in summer. Similar to the growth promotion by vernalization (Stapleton and Jones, 1987), these differences are independent of daylength (Parsons et al., 2013). It is thus likely that epigenetic changes that occur during vernalization determine GA response, but the mechanisms that underlie this interaction have not been explored.

## Approaches for Analyzing the Genetic Basis of the Growth Response of Grass Species

Molecular tools enabling identification of genes and their variants that are responsible for plant growth in response to environmental and developmental factors are now available for model grass species. *B. distachyon* has emerged as a useful model for molecular studies to identify gene function in grasses. *B. distachyon* can easily be genetically modified, and T-DNA mutant collections are available (Thole et al., 2012). However, *B. distachyon* is annual and not adapted to cold climates (Li et al., 2012), which limits its use as model for perennial cool season forage grasses. In contrast, the relative *B. sylvaticum*, combines many of the advantages of *B. distachyon* with perenniality and growth at higher latitudes. It would therefore be an ideal model system to investigate aspects of temperature-dependent growth and seasonality. The ease with which transgenic lines can be created and self-fertility of *Brachypodium* species make them easier to use in functional studies than out-breeding forage grasses.

Association genetics now allows the identification of genetic polymorphisms that determine important traits. In forage grasses, association mapping has so far mainly been limited to candidate genes, e.g., establishing associations between polymorphisms in the *GAI* gene and leaf elongation (Auzanneau et al., 2011) and in a C-repeat binding factor (*CBF*) gene with winter survival (Yu et al., 2015). Further development of the genetic tools and approaches (Kopecký and Studer, 2014) should make genome-wide association studies (GWAS) in forage grasses possible in the near future. The knowledge gained can then be directly used in breeding by marker-assisted selection or genomic selection (Hayes et al., 2013; Grinberg et al., 2016).

## Monitoring Grassland Productivity

Monitoring grassland productivity provides valuable information for scientists, advisors and researchers. There is a long history of recording grassland production through cutting and weighing grass samples in the field (e.g., Davies and Simons, 1979; Binnie et al., 2001; O’Connor et al., 2012). For physiological research that requires recording of subtle differences in grass growth under controlled conditions or in the field, the Leaf Length Tracker (Nagelmüller et al., 2016) provides accurate information for leaf elongation which could, e.g., be used to determine the genetic basis of physiological responses. However, this technology was not developed with the aim of determining biomass production under management regimes such as cutting and grazing.

The small-plot cutting method is the most widely used research technique (Hopkins, 2000) which attempts to simulate grazing or forage conservation management with different intervals, typically of 3 or 4 weeks. There are a range of methods available for measurement. The method developed by Corral and Fenlon (1978) involves recording the yield from four series of plots harvested in rotation, 1 week apart. Grass growth is estimated using a simple quadratic function which accelerates grass growth steadily from zero immediately after harvest.

Hurtado-Uria et al. (2013a) used multiple regression analysis to examine the relationship between grass growth measured using the Corral and Fenlon (1978) methodology and meteorological data at Teagasc, Animal and Grassland Research and Innovation Centre (AGRIC), Moorepark, Fermoy, County Cork in the south of Ireland from 1982 to 2010. Those authors found that the effects of a number of meteorological factors on grass growth varied depending on season (**Table 1**). Temperature has a significant effect in all seasons, as does evapotranspiration.

**Table 1:** Meteorological factors influencing grass growth based on an analysis of grass growth and meteorological conditions recorded at Teagasc, AGRIC, Moorepark, Fermoy, County Cork, Ireland by Hurtado-Uria et al. (2013a).

Season	Variable
(1) January to March	Evapotranspiration
	Soil temperature at 100 mm
(2) April to mid-June	Soil temperature at 50 mm
(3) Mid-June to August	Maximum temperature
	Evapotranspiration
	Minimum temperature
	Sunshine hours
(4) September to December	Evapotranspiration
	Minimum temperature

Plot based estimates of grassland production are useful but somewhat artificial in the context of grass-based ruminant production systems in which grazing comprises the predominant method of feeding. Techniques such as the rising platemeter, cut and weigh, and visual estimation can be used on farm (O’Donovan, 2000). Monitoring grassland production using these techniques provides farmers with reliable information with which to make decisions around managing grass supply, selection of paddocks for grazing or silage, feeding, supplementation, and fertilizer.

Collecting herbage production data from commercial dairy, beef and sheep farms in a database will allow the evaluation of the effects of various management, soil and meteorological factors on grass production. Recently, Teagasc, AGRIC, Moorepark, Fermoy, County Cork, Ireland developed PastureBase Ireland^1^ for this purpose. O’Donovan et al. (2016) used the database to examine the effects of spring nitrogen fertilizer application and autumn closing date on spring herbage production.

Other methods for measuring grassland productivity include remote sensing and eddy covariance analysis. Remote sensing is potentially useful for comparing the current state of grass growth to the average, to the same time the previous year, or between regions. Different methods can be used to analyze satellite images with different levels of accuracy (Ali et al., 2016). The main limitations of remote sensing include the image resolution and noise associated with the satellite observations. Eddy covariance (eddy flux) analysis can be used to monitor grassland CO_2_ exchange. While this method does not provide a direct measure of grass growth, measurement of net ecosystem CO_2_ exchange in intensively managed grassland in Ireland indicated highest productivity in April and May (Peichl et al., 2011), which is in agreement with grass biomass production (Hurtado-Uria et al., 2013a). However, in contrast to foliage production (**Table 1**), net ecosystem CO_2_ exchange was not correlated with spring temperature (Peichl et al., 2011), which indicates differences in the response of leaf growth and below-ground processes to temperature.

## Modeling Grass Growth in a Changing Climate

In the last number of decades many models that describe grass growth have been developed, varying from simple empirical (e.g., Brereton et al., 1996) to more complex mechanistic models (e.g., Thornley, 1998; Jouven et al., 2006; Johnson et al., 2008). Such models provide increased understanding of the processes involved in grass growth and its interaction with farm management, as well as examining the environmental impact on grassland. Grass growth models are often a sub-model or component of larger farm system models, e.g., in APSIM (Keating et al., 2003), PaSim (Soussana et al., 2004; Graux et al., 2011), EcoMod, DairyMod and the SGS Pasture Model (Johnson et al., 2008), and as such are fully integrated with animal intake, grazing behavior and production, as well as N and C balances and other environmental impacts. Integrated models can be used to examine the effects of a changing climate on grass production and the impacts on animal production systems; e.g., Graux et al. (2011) used the PaSim model to assess climate change effects on pasture and herbivore production. Evaluation of models is important to ensure that a model selected for use in a particular region or scenario is useful (Hurtado-Uria et al., 2013b).

User-friendly grass growth prediction models that provide real time information using forecasted meteorological data and farm management data to predict grass growth and hence herbage supply can allow farmers to manage a key resource on their farms. Incorporating machine learning techniques into a grass growth predictor would increase the accuracy of the prediction for individual farms. Combining mechanistic grass growth models with remote sensing analysis of grass production is likely to improve the accuracy of grass growth prediction.

## Consequences for Breeding to Improve Grassland Productivity

While grass breeders have traditionally focused on total annual DM production when breeding grasses, there is an increasing realization that seasonal DM production is important. O’Donovan et al. (2011) highlighted the importance of early spring grass in pasture-based ruminant production systems. McEvoy et al. (2010) reported that winter DM yield is worth up to five times the value of spring and summer DM yield in Irish pasture-based dairy production systems. As highlighted here, a key trait that should be focused on in breeding is overwinter and early spring growth. This will require a focus on the response to environmental and internal (metabolic and hormone) signals in breeding to overcome the conservative utilization of resources that limits growth (Parsons et al., 2011, 2013). However, breeding grasses with increased overwinter growth must be undertaken with care, especially when the grasses are used in areas that experience even occasional cold winters (Stewart and Hayes, 2011). Stewart and Hayes (2011) suggest that it may be possible to combine strong early spring growth with a suitable level of winter hardiness even for colder winter regions (see Cold Acclimation and Winter Persistence).

Advances in plant breeding to meet the requirements for various traits depends on the type and quality of the germplasm/ genetic resources available, and while selecting for a particular trait, breeders have to be aware of the other key plant traits required for the environment and system they are breeding for. Using traditional methods, the timescale for delivering a new grass cultivar to the market is up to 18 years. Adoption of new technologies, such as GWAS and genomic selection (Hayes et al., 2013; Kopecký and Studer, 2014; Grinberg et al., 2016) may increase this rate of delivery, which is vital in view of the current rapid change in climate.

## Author Contributions

AW wrote Section “Introduction, Seasonal Patterns of Grass Growth, Cold Acclimation and Winter Persistence, Physiological Mechanisms Underlying Grassland Productivity During the Growing Season, What Determines the Seasonality of Grass Growth?, Approaches for Analyzing the Genetic Basis of the Growth Response of Grass Species” and coordinated submission of the manuscript. DH wrote Section “Monitoring Grassland Productivity, Modeling Grass Growth in a Changing Climate, Consequences for Breeding to Improve Grassland Productivity” and provided **Table 1**. Both authors read and approved the whole manuscript.

## Conflict of Interest Statement

The authors declare that the research was conducted in the absence of any commercial or financial relationships that could be construed as a potential conflict of interest.

## Footnotes

1 https://www.pasturebase.teagasc.ie/
